# Role of Tissue Hydraulic Permeability in Convection-Enhanced Delivery of Nanoparticle-Encapsulated Chemotherapy Drugs to Brain Tumour

**DOI:** 10.1007/s11095-022-03261-7

**Published:** 2022-04-26

**Authors:** Yi Yang, Wenbo Zhan

**Affiliations:** 1 School of Engineering, King’s College, University of Aberdeen, Aberdeen, AB24 3UE UK; 2 Department of Mechanical Engineering, Imperial College London, London, SW7 3AZ UK

**Keywords:** brain tumour, convection-enhanced delivery, drug transport, mathematical modelling, tissue hydraulic permeability

## Abstract

**Purpose:**

Tissue hydraulic permeability of brain tumours can vary considerably depending on the tissue microstructure, compositions in interstitium and tumour cells. Its effects on drug transport and accumulation remain poorly understood.

**Methods:**

Mathematical modelling is applied to predict the drug delivery outcomes in tumours with different tissue permeability upon convection-enhanced delivery. The modelling is based on a 3-D realistic tumour model that is extracted from patient magnetic resonance images.

**Results:**

Modelling results show that infusing drugs into a permeable tumour can facilitate a more favourable hydraulic environment for drug transport. The infused drugs will exhibit a relatively uniform distribution and cover a larger tumour volume for effective cell killing. Cross-comparisons show the delivery outcomes are more sensitive to the changes in tissue hydraulic permeability and blood pressure than the fluid flow from the brain ventricle. Quantitative analyses demonstrate that increasing the fluid gain from both the blood and brain ventricle can further improve the interstitial fluid flow, and thereby enhance the delivery outcomes. Furthermore, similar responses to the changes in tissue hydraulic permeability can be found for different types of drugs.

**Conclusions:**

Tissue hydraulic permeability as an intrinsic property can influence drug accumulation and distribution. Results from this study can deepen the understanding of the interplays between drug and tissues that are involved in the drug delivery processes in chemotherapy.

## INTRODUCTION

Glioblastoma is the most common primary malignant brain tumour that makes up 16% of all primary brain tumours and 54% of all gliomas (1). The typical survival length is limited to 15 months even if the maximum treatment is applied (2). It is classified as Grade IV by the World Health Organisation as the most aggressive brain cancer. Such a high mortality rate can largely be attributed to the blood–brain barrier (BBB) that can successfully block over 98% of drugs (3) in the bloodstream upon intravenous administration (4). As a development, convection-enhanced delivery (CED) has been developed to bypass this barrier mechanically. Upon CED, anticancer drugs in the infusate are directly infused into the tumour tissue through a catheter (5). This infusion can enhance the bulk movement of interstitial fluid and thereby improve the drug transport for deeper penetration in the tumour.

Brain interstitial fluid (ISF) flow is an essential physiological process that contributes to the nutrient and oxygen supply to cells and waste clearance, due to the lack of functional lymphatics in the brain (6). Fluid from the blood circulatory system is one important source of ISF (7, 8), since the barrier function of the BBB is the result of a highly regulated and complex cellular and molecular transport process (9–11). The ISF also comes from cerebrospinal fluid (CSF). On the one hand, CSF in the subarachnoid space enters the perivascular space on the brain surface (12) and then travels alongside the arteries into the brain parenchyma. On the other hand, the ventricle surface was found with some degrees of permeability which enables cross-ventricle transport (8). Moreover, the ventricle surface becomes highly permeable when hydrocephalus takes place (13, 14). The ISF flow in the brain has been observed in past studies (8). It strongly depends on the tissue hydraulic permeability which stands for the ability of a tissue to enable ISF to transport through the extracellular space. This permeability as an intrinsic tissue property integrates the efforts of multiple factors, including the volume fraction of tissue interstitium, the microstructure and compositions of extracellular matrix, and the arrangement and morphological characteristics of tumour cells (15). How tissue hydraulic permeability influences the delivery outcomes of CED remains unclear.

Drug delivery is composed of multiple physiological and physicochemical processes that are determined by the interplays between the biological properties of the tumour and the transport properties of the drugs (16). Mathematical modelling as a promising tool allows the effects of each influencing factor to be examined individually or in an integrated manner (17, 18). Dividing the entire central nervous system (CNS) into multiple interconnected compartments, the physiologically based pharmacokinetic (PBPK) models were developed to describe the complex transport of drugs between these compartments (19–21). The PBPK models can not only adequately predict the time courses of drug concentration across the CNS compartments but also reveal the role of key factors, such as P-glycoprotein and CSF flow. The results would provide valuable information for developing drugs that target the CNS. Besides, the transport-based model on the macroscale was developed by treating the tumour and its holding tissue as porous media. Consequently, capillary vessels are usually simplified as a distributed source in the model governing equations, avoiding representing the tumour vasculature explicitly. Although drug transport at a single capillary level cannot be taken into account because of this simplification, this model allows accommodating the effects of realistic tumour shape for predicting the spatiotemporal profile of drug concentration in the entire tissue. The modelling framework was firstly established in 1-D in the pioneering studies (22–24) on the delivery of antibodies upon intravenous administration. It was then developed in 3-D and applied to examine the impacts of various tumour biological properties, such as transvascular permeability (25, 26), tumour size and shape (27, 28), and lymphatic drainage (18). A module for simulating drug transport upon pressure-driven fluid infusion was further integrated into the modelling framework for CED. This tailored model was used to investigate the drug transport and accumulation in both the normal brain tissue (29, 30) and brain tumour (31) upon CED. Furthermore, using medical images (32), a commercially available code package iPlanFlow™ (33) has been developed to fast predict the CED delivery outcomes for treatment design.

In this study, a transport-based model is applied to examine the effects of hydraulic permeability of tumour tissue on the performance of nanoparticle-mediated CED. The modelling is based on a 3-D realistic brain tumour model that is reconstructed from the patient Magnetic Resonance (MR) images. The model is designed to capture the key processes in the intracerebral drug delivery, including the CSF and ISF flow, fluid gain from the blood, drug transport by convection with the interstitial fluid flow, drug diffusive transport in the extracellular space, drug release from nanoparticles, drug binding with proteins, elimination due to bioreactions and physical degradation. The treatment is evaluated by the effective distribution volume (${V}_{\mathrm{eff}}$) where the local drug concentration is above the drug LD90.

## MATERIALS AND METHODS

### Mathematical Model

Fluid exchange exists between microcirculation and tissue, determined by the effective transvascular pressure gradient and microvasculature distribution. Given the inter-capillary distance is usually 2 ~ 3 orders lower as compared to the tissue dimension (34), both the brain tumour and its surrounding normal tissue are treated as porous media. The incompressible, Newtonian interstitial fluid flow is governed by the continuity equation and momentum equation, as

1 $$\nabla \bullet \mathbf{v}={F}_{\mathrm{b}}$$

2 $$\rho \left(\frac{\partial \mathbf{v}}{\partial t}+\mathbf{v}\bullet \nabla \mathbf{v}\right)=-\nabla {p}_{\mathrm{i}}+\mu {\nabla }^{2}\mathbf{v}-\frac{\mu }{\kappa }\mathbf{v}$$

where $t$ is time. ${p}_{\mathrm{i}}$ and $\mathbf{v}$ are the interstitial fluid pressure (IFP) and velocity (IFV), respectively. $\rho$ is the density of the interstitial fluid, and $\mu$ is its viscosity. $\kappa$ refers to the tissue hydraulic permeability that can vary significantly depending on the tissue microstructure and compositions. ${F}_{\mathrm{b}}$ is the fluid transporting rate from the blood to tissue, defined by Starling’s law, as

3 $${F}_{\mathrm{b}}={L}_{\mathrm{b}}\frac{S}{V}\left[{p}_{\mathrm{b}}-{p}_{\mathrm{i}}-{\sigma }_{\mathrm{T}}\left({\pi }_{\mathrm{b}}-{\pi }_{\mathrm{i}}\right)\right]$$

in which ${L}_{\mathrm{b}}$ is the hydraulic conductivity of blood vessel wall, $S/V$ is the surface area of blood vessel in a unit tissue volume. ${p}_{\mathrm{b}}$ is the blood pressure. ${\sigma }_{\mathrm{T}}$ stands for the averaged osmotic reflection coefficient for proteins in the blood. ${\pi }_{\mathrm{b}}$ and ${\pi }_{\mathrm{i}}$ are the osmotic pressure of blood and ISF, respectively. Given there is a lack of lymphatic vessels in the brain (6), the fluid loss to the lymphatic circulatory system is not considered.

**Fig. 1:**
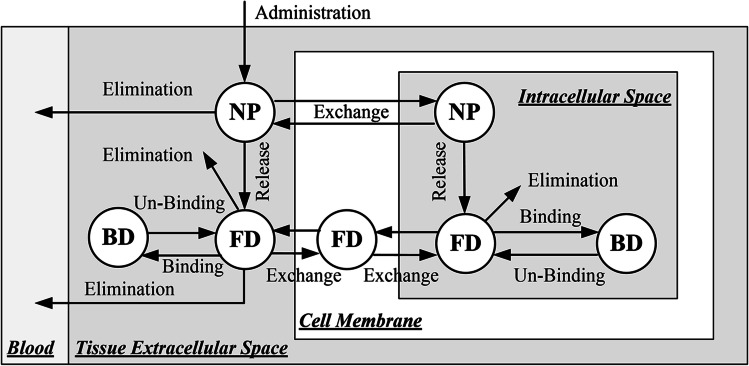
Transport of nanoparticle-mediated drug delivery.

The entire brain including the embedded tumour can be briefly divided into the extracellular space (ECS), cell membrane (CM) and intracellular space (ICS). The transport processes of drugs after being infused are schematically illustrated in Fig. 1. The letters NP, FD and BD refer to the nanoparticles, free drugs and drugs that bind with proteins, respectively. It is assumed that only the drugs in the free form can cross the cell membrane to enter the cell interior (35).

Following the principle of conservation of mass, the concentration of nanoparticles in tissue (${C}_{\mathrm{NP}}$) is governed by

4 $${C}_{\mathrm{NP}}={\varphi }_{\mathrm{ECS}}{C}_{\mathrm{NP},\mathrm{ECS}}+{\varphi }_{\mathrm{CM}}{C}_{\mathrm{NP},\mathrm{CM}}+{\varphi }_{\mathrm{ICS}}{C}_{\mathrm{NP},\mathrm{ICS}}$$

where $\varphi$ is the volume fraction, and ${\varphi }_{\mathrm{CM}}=1-{\varphi }_{\mathrm{ECS}}-{\varphi }_{\mathrm{ICS}}$. It is assumed that no nanoparticle would deposit on the cell membrane, so ${C}_{\mathrm{NP},\mathrm{CM}}$ is set as zero. The transport of nanoparticles in the tissue ECS depends on the diffusive transport driven by the concentration gradient, convective transport with the ISF flow, cell uptake and elimination due to the local drug release and loss to the blood circulatory system. Therefore, the concentration of nanoparticles (${C}_{\mathrm{NP}}$) can be calculated by

5 $$\frac{\partial {C}_{\mathrm{NP}}}{\partial t}={D}_{\mathrm{NP},\mathrm{ECS}}{\nabla }^{2}\left({\varphi }_{\mathrm{ECS}}{C}_{\mathrm{NP},\mathrm{ECS}}\right)-\nabla \bullet \left(\mathbf{v}{\varphi }_{\mathrm{ECS}}{C}_{\mathrm{NP},\mathrm{ECS}}\right)-{k}_{\mathrm{rel}}{\varphi }_{\mathrm{ECS}}{C}_{\mathrm{NP},\mathrm{ECS}}-{k}_{\mathrm{rel}}{\varphi }_{\mathrm{ICS}}{C}_{\mathrm{NP},\mathrm{ICS}}-{k}_{\mathrm{NP},\mathrm{b}}{\varphi }_{\mathrm{ECS}}{C}_{\mathrm{NP},\mathrm{ECS}}$$

where ${D}_{\mathrm{NP},\mathrm{ECS}}$ is the diffusivity of nanoparticles in the tissue ECS. ${k}_{\mathrm{rel}}$ is drug release rate. ${k}_{\mathrm{NP},\mathrm{b}}$ is the rate of nanoparticles transporting to the blood, defined by ${k}_{\mathrm{NP},\mathrm{b}}={P}_{\mathrm{NP}}S/V$. ${P}_{\mathrm{NP}}$ is the nanoparticle transvascular permeability. Hence, Eq. (5) can be written as

6 $$\frac{\partial {C}_{\mathrm{NP},\mathrm{ECS}}}{\partial t}={D}_{\mathrm{NP},\mathrm{ECS}}^{*}{\nabla }^{2}{C}_{\mathrm{NP},\mathrm{ECS}}-{\mathbf{v}}_{\mathrm{NP}}^{*}\bullet \nabla {C}_{\mathrm{NP},\mathrm{ECS}}-{k}_{\mathrm{NP},\mathrm{rel}}^{*} {C}_{\mathrm{NP},\mathrm{ECS}}-{k}_{\mathrm{NP},\mathrm{clr}}^{*}{C}_{\mathrm{NP},\mathrm{ECS}}$$

where ${D}_{\mathrm{NP},\mathrm{ECS}}^{*}={\varphi }_{\mathrm{ECS}}{D}_{\mathrm{NP},\mathrm{ECS}}/{h}_{\mathrm{NP}}$ is the apparent diffusivity of nanoparticles in the tissue ECS, and ${\mathbf{v}}_{\mathrm{NP}}^{*}={\varphi }_{\mathrm{ECS}}\mathbf{v}/{h}_{\mathrm{NP}}$ is the apparent IFV. ${k}_{\mathrm{NP},\mathrm{rel}}^{*}={{k}_{\mathrm{NP},\mathrm{rel}}/{h}_{\mathrm{NP}}=k}_{\mathrm{rel}}\left({\varphi }_{\mathrm{ECS}}+{\varphi }_{\mathrm{ICS}}{P}_{\mathrm{NP},\mathrm{ICS}-\mathrm{ECS}}\right)/{h}_{\mathrm{NP}}$ is the apparent drug release rate. ${k}_{\mathrm{NP},\mathrm{clr}}^{*}={k}_{\mathrm{NP},\mathrm{clr}}/{h}_{\mathrm{NP}}=\left({\varphi }_{\mathrm{ECS}}{k}_{\mathrm{NP},\mathrm{b}}+{F}_{\mathrm{b}}\right)/{h}_{\mathrm{NP}}$ is the apparent elimination rate of nanoparticles. ${h}_{\mathrm{NP}}={\varphi }_{\mathrm{ECS}}+{\varphi }_{\mathrm{ICS}}{P}_{\mathrm{NP},\mathrm{ICS}-\mathrm{ECS}}$ is determined by the properties of the nanoparticle and tissue. Given both the tissue ECS and ICS are aquatic phases, the partition coefficient between these two compartments (${P}_{\mathrm{NP},\mathrm{ICS}-\mathrm{ECS}}$) is assumed to be unity (36).

Similarly, the concentrations of free drugs (${C}_{\mathrm{FD}}$) and bound drugs (${C}_{\mathrm{BD}}$) in tissues are governed by

7 $$\begin{array}{c}{C}_{\mathrm{FD}}={\varphi }_{\mathrm{ECS}}{C}_{\mathrm{FD},\mathrm{ECS}}+{\varphi }_{\mathrm{CM}}{C}_{\mathrm{FD},\mathrm{CM}}+{\varphi }_{\mathrm{ICS}}{C}_{\mathrm{FD},\mathrm{ICS}}\\ {C}_{\mathrm{BD}}={\varphi }_{\mathrm{ECS}}{C}_{\mathrm{BD},\mathrm{ECS}}+{\varphi }_{\mathrm{CM}}{C}_{\mathrm{BD},\mathrm{CM}}+{\varphi }_{\mathrm{ICS}}{C}_{\mathrm{BD},\mathrm{ICS}}\end{array}$$

Since there is a lack of bound drugs that would deposit on the cell membrane, ${C}_{\mathrm{BD},\mathrm{CM}}$ is assumed to be zero (36). The transport of free drugs in the tissue ECS is determined by convection and diffusion, elimination due to bioreactions, degradation, loss to the blood circulatory system and binding with proteins. The concentration of free drugs (${C}_{\mathrm{FD}}$) can be calculated by

8 $$\frac{\partial {C}_{\mathrm{FD}}}{\partial t}={D}_{\mathrm{FD},\mathrm{ECS}}{\nabla }^{2}\left({\varphi }_{\mathrm{ECS}}{C}_{\mathrm{FD},\mathrm{ECS}}\right)-\nabla \bullet \left(\mathbf{v}{\varphi }_{\mathrm{ECS}}{C}_{\mathrm{FD},\mathrm{ECS}}\right)-{\varphi }_{\mathrm{ECS}}\left({k}_{\mathrm{FD},\mathrm{b}}+{k}_{\mathrm{FD},\mathrm{e}}\right){C}_{\mathrm{FD},\mathrm{ECS}}-{\varphi }_{\mathrm{ICS}}{k}_{\mathrm{FD},\mathrm{e}}{C}_{\mathrm{FD},\mathrm{ECS}}+{k}_{\mathrm{rel}}{\varphi }_{\mathrm{ECS}}{C}_{\mathrm{NP},\mathrm{ECS}}+{k}_{\mathrm{rel}}{\varphi }_{\mathrm{ICS}}{C}_{\mathrm{NP},\mathrm{ICS}}-\frac{\partial {C}_{\mathrm{BD}}}{\partial t}$$

in which ${D}_{\mathrm{FD},\mathrm{ECS}}$ is the diffusivity of free drugs in the tissue ECS. ${k}_{\mathrm{FD},\mathrm{b}}$ is the rate of drug loss to the blood, and ${k}_{\mathrm{FD},\mathrm{e}}$ is the elimination rate combining the contributions of bioreactions and physical degradation. Two assumptions are further introduced. Firstly, since the dynamic process of drug binding with proteins takes place on a smaller time scale as compared to drug transport, the concentration of free drugs and bound drugs are linearly correlated (37, 38), as ${K}_{\mathrm{ECS}}={C}_{\mathrm{BD},\mathrm{ECS}}/{C}_{\mathrm{FD},\mathrm{ECS}}$ and ${K}_{\mathrm{ICS}}={C}_{\mathrm{BD},\mathrm{ICS}}/{C}_{\mathrm{FD},\mathrm{ICS}}$. Secondly, the concentration of free drugs reaches dynamic equilibrium between ECS, CM and ICS (39, 40), as ${P}_{\mathrm{FD},\mathrm{ICS}-\mathrm{ECS}}={C}_{\mathrm{FD},\mathrm{ICS}}/{C}_{\mathrm{FD},\mathrm{ECS}}$ and ${P}_{\mathrm{CM}-\mathrm{ECS}}={C}_{\mathrm{FD},\mathrm{CM}}/{C}_{\mathrm{FD},\mathrm{ECS}}$. Therefore, Eq. (8) can be simplified as

9 $$\frac{\partial {C}_{\mathrm{FD},\mathrm{ECS}}}{\partial t}={D}_{\mathrm{FD},\mathrm{ECS}}^{*}{\nabla }^{2}{C}_{\mathrm{FD},\mathrm{ECS}}-{\mathbf{v}}_{\mathrm{FD}}^{*}\bullet \nabla {C}_{\mathrm{FD},\mathrm{ECS}}-{k}_{\mathrm{FD},\mathrm{clr}}^{*}{C}_{\mathrm{FD},\mathrm{ECS}}+{k}_{\mathrm{FD},\mathrm{rel}}^{*}{C}_{\mathrm{NP},\mathrm{ECS}}$$

where ${D}_{\mathrm{FD},\mathrm{ECS}}^{*}={\varphi }_{\mathrm{ECS}}{D}_{\mathrm{FD},\mathrm{ECS}}/{h}_{\mathrm{FD}}$ is the apparent diffusivity of free drugs in the tissue ECS, and ${\mathbf{v}}_{\mathrm{FD}}^{*}={\varphi }_{\mathrm{ECS}}\mathbf{v}/{h}_{\mathrm{FD}}$ is the apparent IFV. ${k}_{\mathrm{FD},\mathrm{rel}}^{*}={k}_{\mathrm{rel}}\left({\varphi }_{\mathrm{ECS}}+{\varphi }_{\mathrm{ICS}}{P}_{\mathrm{NP},\mathrm{ICS}-\mathrm{ECS}}\right)/{h}_{\mathrm{FD}}$ is the apparent drug release rate. ${k}_{\mathrm{FD},\mathrm{clr}}^{*}={k}_{\mathrm{FD},\mathrm{clr}}/{h}_{\mathrm{FD}}=\left[{\varphi }_{\mathrm{ECS}}{k}_{\mathrm{FD},\mathrm{b}}+\left({\varphi }_{\mathrm{ECS}}+{\varphi }_{\mathrm{ICS}}\right){k}_{\mathrm{FD},\mathrm{e}}+{F}_{\mathrm{b}}\right]/{h}_{\mathrm{FD}}$ is the apparent elimination rate of free drugs. ${h}_{\mathrm{FD}}={\varphi }_{\mathrm{ECS}}\left(1+{K}_{\mathrm{ECS}}\right)+{\varphi }_{\mathrm{ICS}}{P}_{\mathrm{ICS}-\mathrm{ECS}}\left(1+{K}_{\mathrm{ICS}}\right)+{\varphi }_{\mathrm{CM}}{P}_{\mathrm{CM}-\mathrm{ECS}}$ is determined by the properties of the drug and tissue.

### Model Geometry

**Fig. 2:**
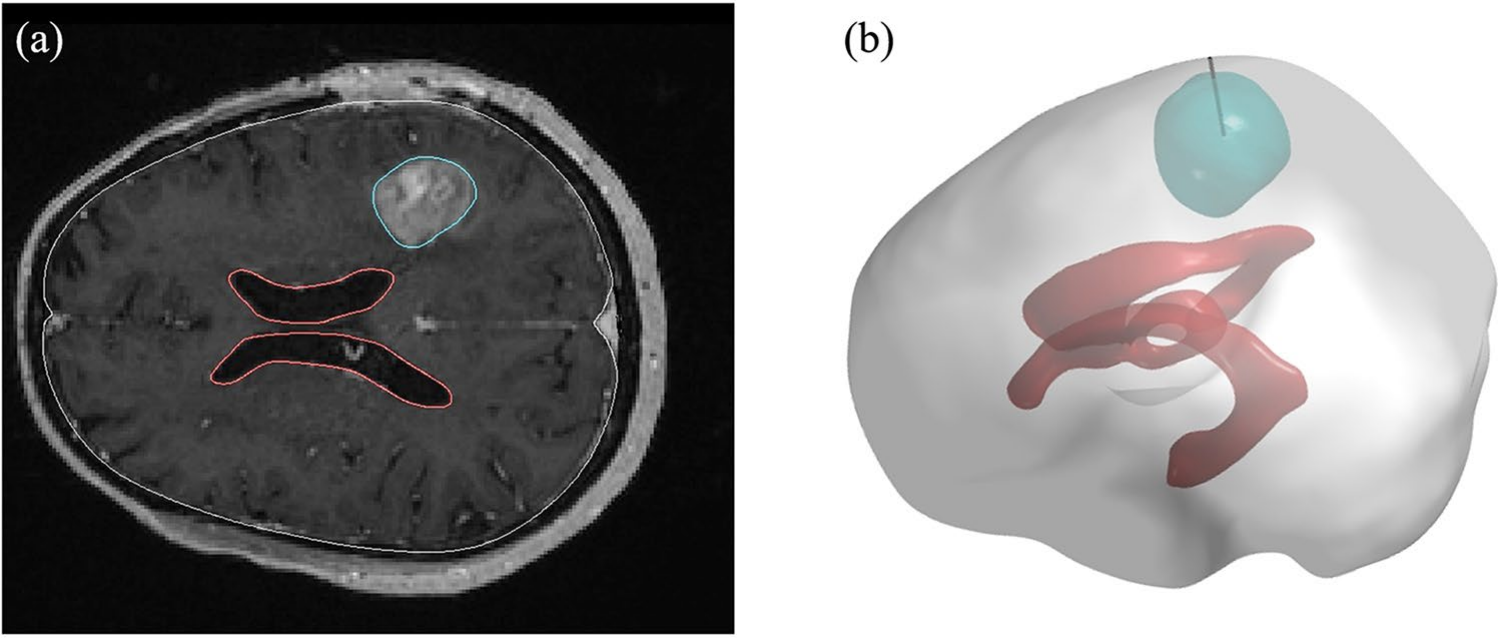
Model geometry. (**a**) The representative slice of MR images used for geometry reconstruction, (**b**) the reconstructed 3-D geometry

The 3-D geometry of a brain including the embedded tumour is reconstructed from a set of patient MR images, which is available on the image database TCIA under the Creative Commons Attribution 3.0 Unported license for scientific purpose (41, 42). These images were acquired in three orthogonal planes and anonymised. Each image slice comprises 256 by 256 1-mm pixels and is 1 mm thick. A representative slice used in this study is given in Fig. 2(a).

The brain tumour and its holding tissue are segmented on each MR image slice based on the local signal intensity. The resulting surfaces of the tumour, brain tissue and ventricle are firstly smoothed and then imported into the meshing software ANSYS ICEM CFD (ANSYS Inc., Canonsburg, USA). The mesh independence test is performed, and the final computational mesh consists of 5.1 million tetrahedral elements. The volumes of brain normal tissue and tumour are measured as 1387 and 25 cm^3^, respectively. Drugs are delivered through a 1.0 mm-diameter catheter, as shown in Fig. 2(b) in black.

### Model Parameters

Since the time window of drug delivery is usually much smaller as compared to the rate of tumour growth, the brain and drug properties are assumed to be time-independent. Temozolomide is selected as a representative drug for brain tumour treatment. The values of the model parameters are summarised in Tables I and II. The justification for selecting the key parameters is given below.

**Table I:** Parameters for the Tissues.

Symbol	Parameter	Brain tumour	Normal tissue	Source
$\rho$	Density of interstitial fluid (kg/m^3^)	1000	1000	(43)
$\mu$	Viscosity of interstitial fluid (kg/m/s)	7.8E-4	7.8E-4	(43)
${\pi }_{\mathrm{b}}$	Osmotic pressure of blood (Pa)	3440	3440	(44)
${\pi }_{\mathrm{i}}$	Osmotic pressure of interstitial fluid (Pa)	1110	740	(22)
${p}_{\mathrm{b}}$	Pressure of blood (Pa)	4610	4610	(44)
$S/V$	Surface area of blood vessels per tissue volume (m^−1^)	20,000	7000	(22)
${\sigma }_{\mathrm{T}}$	Osmotic reflection coefficient for blood proteins (-)	0.82	0.91	(22)
${L}_{\mathrm{b}}$	Hydraulic conductivity of the vessel wall (m/Pa/s)	1.1E-12	1.4E-13	(36)
$\kappa$	Tissue hydraulic permeability (m^2^)	1.0E-15	1.0E-16	(45)

**Table II:** Parameters for the Nanoparticle and Chemotherapy Drugs.

Symbol	Parameter	Nanoparticle	Doxorubicin	Temozolomide	Paclitaxel	Carmustine
*MW*	Molecular weight (g/mol)	-	543.52 (46)	194.15 (47)	853.91 (48)	214.05 (49)
${P}_{\mathrm{ICS}-\mathrm{ECS}}$	Partition coefficient between ICS and ECS (-)	1.0 (36)	1.0 (50)	1.0 (50)	1.0 (50)	1.0 (50)
${P}_{\mathrm{CM}-\mathrm{ECS}}$	Partition coefficient between CM and ECS (-)	-	0.3 (51)	1.5E-2 (52)	3162.3 (53)	10.3 (50)
${K}_{\mathrm{ECS}}$*,* ${K}_{\mathrm{ICS}}$	Binding constant between FD and BD (-)	-	3.0 (54)	1.8E-1 (55)	5.1 (56)	5.0 (50)
${D}_{\mathrm{ECS},\mathrm{t}}$	Diffusion coefficient in extracellular space in tumour (m^2^/s)	9.0E-12 (57)	3.4E-10 (16)	7.2E-10 (58)	9.0E-10 (53)	1.5E-9 (50)
${D}_{\mathrm{ECS},\mathrm{n}}$	Diffusion coefficient in extracellular space in normal tissue (m^2^/s)	5.8E-12 (59)	1.6E-10 (16)	3.4E-10 (60)	1.1E-10 (16)	3.2E-10 (16)
${P}_{\mathrm{b},\mathrm{t}}$	Transvacular permeability in tumour (m/s)	3.4E-9 (59)	0.0 (61)	8.0E-8 (60)	7.0E-9 (53)	7.0E-7 (50)
${P}_{\mathrm{b},\mathrm{n}}$	Transvacular permeability in tumour (m/s)	0.0 (59)	0.0 (61)	4.3E-8 (60)	2.0E-8 (16)	2.0E-6 (16)
${k}_{e}$	Drug elimination due to enzymatic/non-enzymatic reactions (s^−1^)	-	5.8E-4 (18)	1.1E-4 (62)	6.8E-7 (53)	1.1E-4 (50)
${k}_{\mathrm{rel}}$	Release rate (s^−1^)	1.0E-4 (63)	-	-	-	-
${R}_{\mathrm{in}}$	Infusion rate (μL/min)	5.0 (64)	-	-	-	-
${C}_{\mathrm{in}}$	Infusate concentration of the nanoparticle-encapsulated form drugs (mg/mL)	1.0 (64)	-	-	-	-
${C}_{\mathrm{eff}}$	Drug concentration to kill 90% of cells, LD90 (M)	-	2.4E-6 (65)	3.9E-5 (66)	8.9E-7 (53)	1.5E-5 (53)

The subscript $\mathrm{t}$ refers to tumour, and $\mathrm{n}$ refers to normal tissue.

#### Tissue Hydraulic Permeability ($\it \upkappa$)

The permeability of normal brain tissue was measured on a scale of 1.0E-16 m^2^ (45). The value could vary considerably in tumours. The permeability of human glioblastoma was reported as 4.9E-16 m^2^ (67). Moreover, a correlation between the tissue hydraulic permeability and glycosaminoglycans (15) suggests that $\kappa$ ranges from 1.3E-14 to 9.1E-14 m^2^. To cover the potential levels the tissue hydraulic permeability can reach in brain tumours, a large range from 1.0E-16 to 1.0E-13 m^2^ is applied in this study. The baseline value is set as 1.0E-15 m^2^.

#### Infusion Rate (${\mathrm{R}}_{\mathrm{in}}$)

The infusion rate can be precisely controlled in clinical practice using a syringe pump. It is commonly set in the range of 0.5 ~ 10.0 μL/min (64, 68) to avoid possible tissue damage (69). The catheter can be left indwelling for several days when the infusion rate is kept below 5.0 μL/min (70). Since 5.0 μL/min has been applied in the clinical trials (64), the same infusion rate is used in this study.

#### Infusate Concentration (${\mathrm{C}}_{\mathrm{in}}$)

Infusate concentration directly relates to the administrated dose. Since CED using nanoparticles has yet been applied as a mainstream treatment in clinical practice, there is a lack of references suggesting this concentration. In the clinical trials (64) where plain paclitaxel (PTX) was applied, the infusate concentration was set as 1.0 mg/mL which was over 100-fold higher as compared to the PTX solubility in water (71). This indicates that the PTX suspension rather than the solution was used. Nanoparticles can be extremely soluble, subject to the formulation, particularly the ligands attached to the nanoparticle surface (72, 73). Furthermore, multiple drug molecules can be loaded into a single nanoparticle. These features enable the infusate concentration of nanoparticles to span a large range. Therefore, the infusate concentration of 1.0 mg/mL is also used in this modelling study.

#### Volume Fraction of Tissue Compartment ($\mathrm{\varphi }$)

The volume fractions of each tissue compartment differ significantly depending on the cell type. Normal brain tissue is mainly composed of neurons and glial cells, with the latter being further divided into microglia, astrocytes and oligodendrocytes. Given the total cell ${\varphi }_{\mathrm{ICS}}$ in normal brain tissue was measured as 0.65 (50, 74), the ${\varphi }_{\mathrm{ICS}}$ for each type of cells can be estimated based on the cell population ratio and cell body dimension, as summarised in Table III. The volume fraction of cell membrane (${\varphi }_{\mathrm{CM}}$) in brain tissue strongly depends on the cell dendrites and axons. However, even for the same type of cells, the morphological characteristics of these protoplasmic protrusions, such as length and diameter, can vary significantly from cell to cell. This makes it less feasible to differentiate the ${\varphi }_{\mathrm{CM}}$ for each cell type. Therefore, the ${\varphi }_{\mathrm{ECS}}$ and total ${\varphi }_{\mathrm{CM}}$ of brain normal tissue are set as 0.15 and 0.20 (50, 74), respectively. On the other hand, glioblastoma consists mainly of cancer stem cells. The ${\varphi }_{\mathrm{ICS}}$ is measured as 0.55, while the ${\varphi }_{\mathrm{CM}}$ is around 0.10 (50, 74).

**Table III:** Properties of Different Cells in the Brain.

Cell type	Ratio of cell population (%)*	Cell body diameter ($\mathrm{\mu m}$)	Volume fraction of ICS (-)†
Neuron	50 (75)	4 ~ 24 (76)	6.28E-1
Oligodendrocytes	22.5 ~ 37.5 (77)	6 ~ 8 (78)	7.35E-3
Astrocytes	9.5 ~ 20 (77)	10 ~ 12 (78)	1.43E-2
Microglia	5.0 (77)	2.2 (78)	3.04E-4

* the population ratio between neuron and glial cells is $1:1$ (75).

† the average values of cell population ratio and cell body diameter are used for this estimation.

#### Release Rate (${\mathrm{k}}_{\mathrm{rel}}$)

The release rate refers to the time scale for nanoparticles to release the payload. Depending on the formulation and environment, this property directly determines the therapeutic activities and toxicity of the drug delivery system (79–81). Experiments showed that temperature-sensitive nanoparticles can release the drugs in a few seconds (82), whereas the release would last for weeks for stealth nanoparticles (83). As the corresponding release rates are calculated (84) in the range from 1.0E-1 to 1.0E-6 s^−1^, the value of 1.0E-4 s^−1^ is selected for the modelling in this study.

### Numerical Methods

The mathematical model is implemented in a Computational Fluid Dynamics code package, ANSYS FLUENT (ANSYS Inc., Canonsburg, USA) for generating the numerical solutions. The 2^nd^ order UPWIND scheme is employed for spatial discretisation, and temporal discretisation is achieved using the 2^nd^ implicit Euler scheme. Pressure is related to velocity correction by the SIMPLEC algorithm. The residual tolerance is set as 1.0E-4 to control the modelling convergence. The model for the interstitial fluid flow is solved in the first place. The obtained IFP and IPV are then applied as input for predicting the drug transport and accumulation. All the concentrations are assumed to be zero in the entire brain at the beginning of drug delivery.

### Boundary Conditions

The pressure on the brain surface and ventricle surface are specified as 1447 Pa (44) and 658 Pa (85), respectively. A continuous condition is imposed on the interface between the tumour and its holding tissue. The wall of the infusion catheter is assumed to be rigid with zero flux. The constant flow rate and infusate concentration are applied to the infusion catheter tip for drug administration.

### Quantification of Delivery Outcomes

The delivery outcomes of nanoparticle-encapsulated drugs under different conditions are evaluated from the perspectives of drug accumulation, drug spatial distribution and potential treatment effectiveness. These are represented by quantitative indexes defined below.

#### Spatial-Averaged Concentration

Drug accumulation is determined by the convective and diffusive transport, elimination due to blood drainage, drug release for nanoparticles, and degradation and bioreactions for free chemotherapy drugs. The spatial-averaged drug concentration (${C}_{\mathrm{ECS},\mathrm{avg}}$) is therefore used to evaluate the drug accumulation in the entire tissue, as

10 $${C}_{\mathrm{ECS},\mathrm{avg}}=\frac{\sum {C}_{\mathrm{ECS},\mathrm{i}}{V}_{\mathrm{i}}}{\sum {V}_{\mathrm{i}}}=\frac{\sum {C}_{\mathrm{ECS},\mathrm{i}}{V}_{\mathrm{i}}}{{V}_{\mathrm{tissue}}}$$

where ${C}_{\mathrm{ECS},\mathrm{i}}$ and ${V}_{\mathrm{i}}$ are the local drug concentration and local tissue volume, respectively. ${V}_{\mathrm{tissue}}$ is the volume of the entire tissue.

#### Distribution Nonuniformity

The nonuniformity of drug spatial distribution can be represented by a dimensionless number, $\mathrm{NUN}$, as

11 $$\mathrm{NUN}=\frac{\sum \left|{C}_{\mathrm{ECS},\mathrm{i}}-{C}_{\mathrm{ECS},\mathrm{avg}}\right|{V}_{\mathrm{i}}}{{C}_{\mathrm{ECS},\mathrm{avg}}\sum {V}_{\mathrm{i}}}=\frac{\sum \left|{C}_{\mathrm{ECS},\mathrm{i}}-{C}_{\mathrm{ECS},\mathrm{avg}}\right|{V}_{\mathrm{i}}}{{C}_{\mathrm{ECS},\mathrm{avg}}{V}_{\mathrm{tissue}}}$$

$\mathrm{NUN}$ is the sum of the distances between the local drug mass and the average mass of whole tissue. A higher value indicates a more heterogeneous distribution.

#### Effective Distribution Volume

The treatment is evaluated by the drug's effective distribution volume (${V}_{\mathrm{eff}}$), where the drug extracellular concentration is greater than the drug LD90, as

12 $${V}_{\mathrm{eff}}=\sum {V}_{\mathrm{i}} \left({C}_{\mathrm{ECS},\mathrm{i}}\ge \mathrm{LD}90\right)$$

${V}_{\mathrm{eff}}$ stands for the region where there are adequate drugs for cell killing. A higher value of ${V}_{\mathrm{eff}}$ suggests a more effective treatment.

## RESULTS

### Baseline Study

**Fig. 3:**
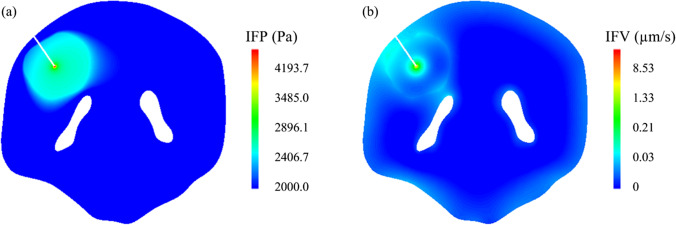
The interstitial fluid flow in the brain tumour and its surrounding tissue (***κ*** = 1.0E-15 m^2^). (**a**) interstitial fluid pressure, and (**b**) interstitial fluid velocity.

Tissue hydraulic permeability as an intrinsic property reflects the tissue resistance to the ISF flow. In this modelling study, the flow field is obtained by solving the governing equations in the whole brain including the embedded tumour, subject to the model parameters in Table I and the aforementioned boundary conditions. As a representative, the ISF flow in the tumour with the baseline tissue hydraulic permeability is represented at a transverse plane in Fig. 3. Results show that the predicted IFP in the tumour is higher than the surrounding normal tissue, which is consistent with the findings from experiments (86). This phenomenon can be attributed to the abnormal properties of the tumour, including the enlarged microvasculature density, increased hydraulic conductivity of blood vessel wall and raised osmotic pressure of the ISF. However, CED can introduce even higher pressure at the infusion site. The resulting pressure gradient from the catheter tip to the tumour surface enhances the ISF flow that delivers the drugs to deeper tumour regions. In addition, IFV is also high at the tumour-normal tissue interface. This is mainly due to a sharp fall in IFP that causes ISF to flow from the tumour to its holding tissue.

Interstitial fluid flow in the brain tumours with different tissue hydraulic permeability are compared in Table IV. Results show that the IFP becomes lower in the permeable tumour due to the lower tissue resistance. This allows for the rapid interstitial fluid flow that can enhance the drug convective transport for deeper penetration. On the other hand, more fluid can transport from the blood into the tissue ECS of the permeable tumour, as indicated by *F*_b_. This fluid gain also aids in drug transport and distribution.

**Table IV:** The Effects of Tissue Hydraulic Permeability on Interstitial Fluid Flow in the Tumour.

*κ* (m^2^)	IFP (Pa)	IFV (nm/s)	*F*_b_ (μs^−1^)
1.0E-13	2549.9	31.7	3.29
1.0E-14	2551.3	30.1	3.26
1.0E-15	2561.5	22.4	3.03
1.0E-16	2594.4	9.2	2.31

**Fig. 4:**
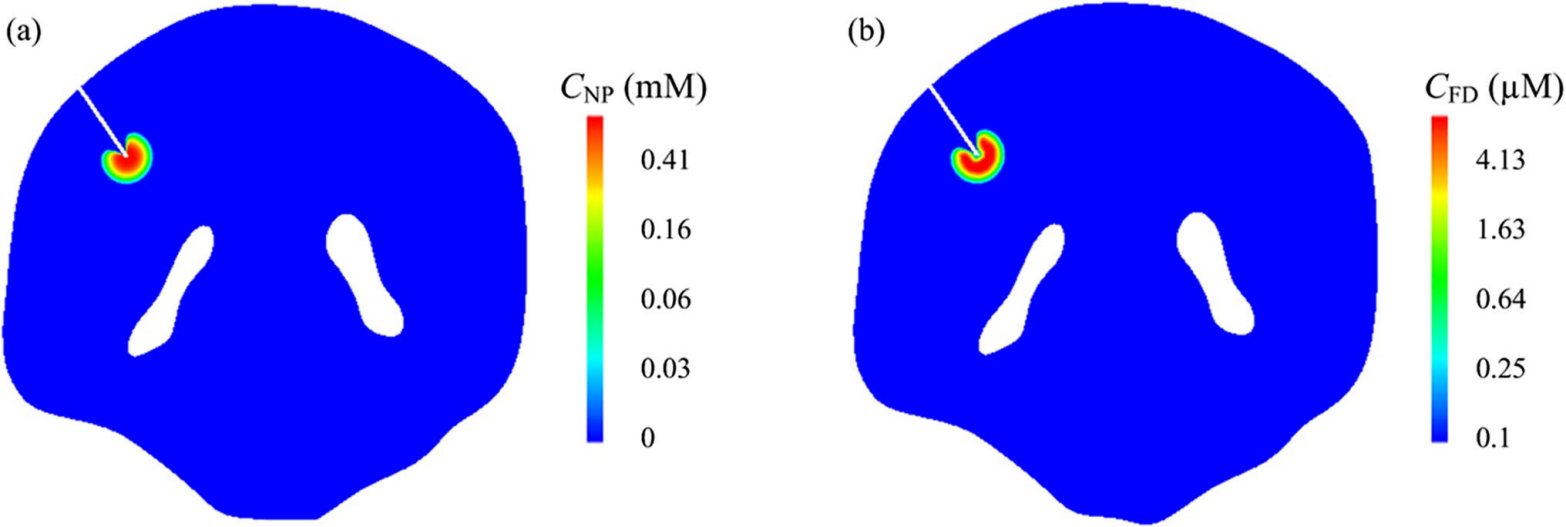
The spatial distribution of drugs on a transverse plane in the brain (***κ*** = 1.0E-15 m^2^). (**a**) nanoparticle-encapsulated temozolomide, and (**b**) released free temozolomide.

Since drugs are infused into the tumour ECS in the nanoparticle-encapsulated form, the concentration and distribution of nanoparticles can have a direct influence on the treatment. The drug spatial distributions at a transverse plane are represented in Fig. 4. It is not surprising that the nanoparticle concentration reaches its peak at the infusion site and decreases radially towards the brain surface. A quantitative analysis shows that this concentration reduces to 1.0‰ of ${C}_{\mathrm{in}}$ in approximately 5 mm away from the catheter tip. A similar distribution pattern can be found for free temozolomide because all the free drugs are released from nanoparticles. These distribution patterns denote that CED would lead to a highly localised delivery outcome. It is beneficial for achieving a precise delivery to minimise the risks of side effects, which are caused by the high drug concentration in normal tissue. However, the treatment of large tumours with a single infusion catheter can be disappointing.

Keeping the infusion settings identical, simulations are run to examine how the hydraulic permeability of tumour tissue influences the delivery outcomes, with results summarised in Table V. Comparisons show that a higher spatial-averaged drug concentration can be achieved in the less permeable tumour. However, these drugs would accumulate in limited regions, as indicated by $\mathrm{NUN}$, resulting in a smaller distribution volume in which tumour cells can be effectively killed. On the contrary, the concentration in the normal tissue is positively related to the hydraulic permeability of tumour tissue. This is because fewer drugs can escape the tumour when the flow resistance is high. Moreover, the concentrations in neurons, oligodendrocytes, astrocytes and microglia are several orders of magnitude lower compared to the tumour cells. This further demonstrates that the delivery outcomes of CED are highly localised. Therefore, the following studies will be focused on brain tumours.

**Table V:** The Effect of Tissue Hydraulic Permeability on Drug Delivery Outcome

Parameter	*κ* = 1.0E-13 (m^2^)	*κ* = 1.0E-14 (m^2^)	*κ* = 1.0E-15 (m^2^)	*κ* = 1.0E-16 (m^2^)
Averaged concentration of NP in tumour cells, ${C}_{\mathrm{avg},\mathrm{NP}} (\mathrm{M})$	9.99E-3	1.01E-2	1.07E-2	1.46E-2
Averaged FD concentration in tumour cells,${C}_{\mathrm{avg},\mathrm{FD}} (\mathrm{M})$	1.62E-4	1.64E-4	1.76E-4	2.43E-4
Non-uniformity of FD in tumour, $\mathrm{NUN}$ ($-$)	1.88	1.88	1.89	1.92
Effective distribution in tumour,${V}_{\mathrm{eff}} ({\mathrm{cm}}^{3})$	1.58	1.56	1.45	1.03
Averaged NP concentration in neurons,${C}_{\mathrm{avg},\mathrm{FD}} (\mathrm{M})$	5.99E-22	1.20E-22	1.63E-28	1.56E-35
Averaged NP concentration in Oligodendrocytes,${C}_{\mathrm{avg},\mathrm{NP}} (\mathrm{M})$	7.01E-24	1.40E-24	1.91E-30	1.56E-35
Averaged NP concentration in Astrocytes,${C}_{\mathrm{avg},\mathrm{NP}} (\mathrm{M})$	1.36E-23	2.73E-24	3.72E-30	7.53E-39
Averaged NP concentration in Microglia,${C}_{\mathrm{avg},\mathrm{NP}} (\mathrm{M})$	2.90E-25	5.80E-26	7.53E-39	7.53E-39
Averaged FD concentration in neurons,${C}_{\mathrm{avg},\mathrm{FD}} (\mathrm{M})$	5.85E-23	1.63E-23	1.39E-29	2.03E-36
Averaged FD concentration in Oligodendrocytes,${C}_{\mathrm{avg},\mathrm{FD}} (\mathrm{M})$	6.84E-25	1.91E-25	1.63E-31	2.03E-36
Averaged FD concentration in Astrocytes,${C}_{\mathrm{avg},\mathrm{FD}} (\mathrm{M})$	1.33E-24	3.72E-25	3.17E-31	9.82E-40
Averaged FD concentration in Microglia,${C}_{\mathrm{avg},\mathrm{FD}} (\mathrm{M})$	2.83E-26	7.90E-27	9.82E-40	9.82E-40

### Cross-Influence with Fluid Gain from Blood

Fluid gain from blood (${F}_{\mathrm{b}}$) as one source of the intracerebral ISF is related to the effective transvascular pressure gradient in which the microvasculature blood pressure (${p}_{\mathrm{b}}$) plays a key role, as defined in Eq. (3). Since ${p}_{b}$ was measured in the range from 2860 to 5333 Pa (44), this range is applied to control ${F}_{\mathrm{b}}$ for considering its impact on the delivery outcomes.

**Fig. 5:**
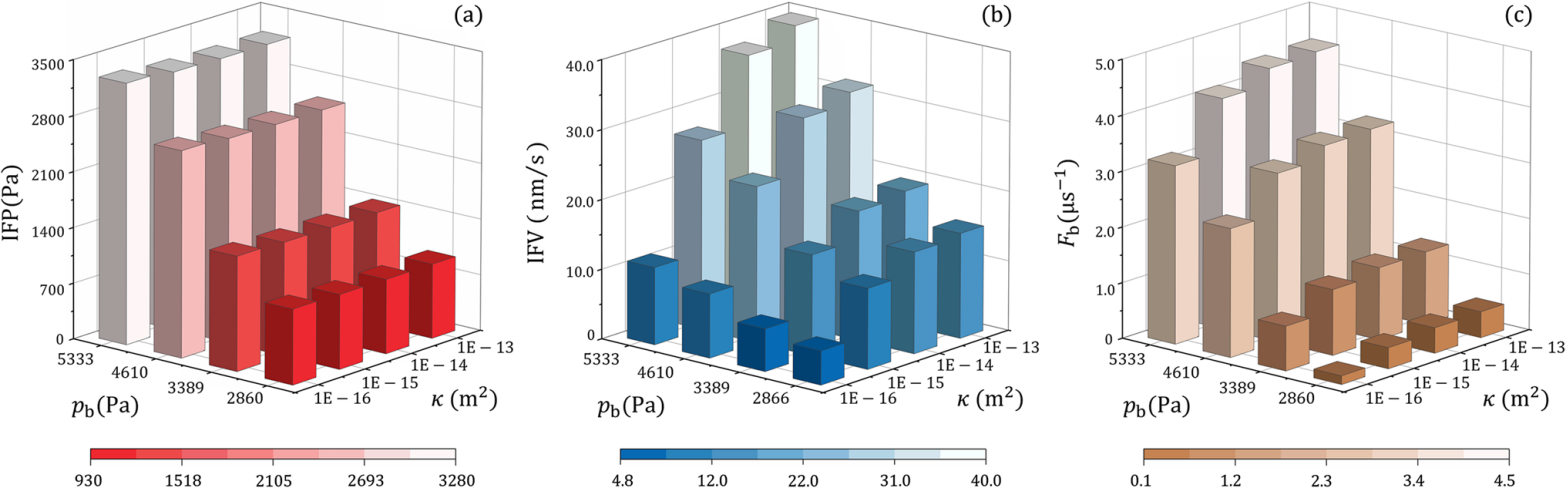
Effect of tissue permeability on the interstitial fluid flow in brain tumours with different blood pressure. (**a**) Interstitial fluid pressure, (**b**) interstitial fluid velocity, and (**c**) fluid gain from blood.

Figure 5 represents the intratumoural hydraulic environments when infusing drugs into brain tumours with different tissue hydraulic permeability and blood pressure. Although IFP presents an inverse relationship with tissue hydraulic permeability as shown in Table IV, comparisons in Fig. 5(a) denote that IFP is more sensitive to blood pressure, and a positive relationship exists. To be different, IFV in Fig. 5(b) increases with tissue permeability and blood pressure. The fastest flow takes place in the tumour with the highest tissue hydraulic permeability and blood pressure. The same pattern can be found for the fluid gain from the blood, as indicated by the results in Fig. 5(c).

**Fig. 6:**
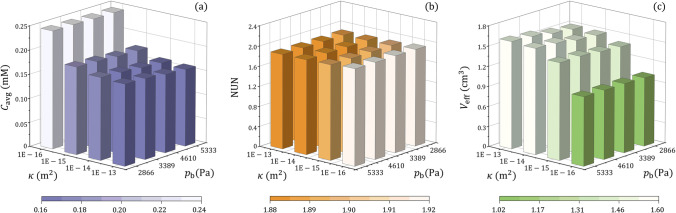
Effect of tissue permeability on the delivery outcomes in brain tumours with different blood pressure. (**a**) Spatial-averaged concentration, (**b**) non-uniformity, and (**c**) effective distribution volume.

The drug delivery outcomes in brain tumours with different tissue permeability and blood pressure are compared in Fig. 6. Results show that the drug concentration in a tumour is mainly determined by tissue hydraulic permeability rather than blood pressure. However, both these two tumour properties have limited impacts on drug distribution. A slightly uniform distribution can be found in the tumour with the highest tissue permeability and blood pressure. This is owing to the highest IFV in this tumour, as shown in Fig. 5(b), enabling the most effective convective drug transport for the deepest penetration. Furthermore, the effective distribution volume is positively related to tissue hydraulic permeability and blood pressure. As a result, better treatment can be achieved in permeable tumours where blood pressure in the microvasculature is also high.

### Cross-Influence with Cerebrospinal Fluid from the Ventricle

CSF is possible to pass across the surface of the ventricle into the brain parenchyma, thereby contributing to intracerebral ISF perfusion. This flow strongly depends on the permeability of the ventricle surface which has yet been accurately measured. In this study, the contribution of the CSF is controlled by the boundary condition imposed on the ventricle surface. Specifically, the local flow flux is set to zero for the impermeable ventricle surface; whereas the local pressure is directly assigned as the ventricle pressure of 1447 Pa (44) when the ventricle surface is fully permeable. The latter ultimately results in a flow of 1.0E-7 kg/s at the ventricle surface, as predicted by the modelling. Therefore, two other flow fluxes, 6.5E-8 kg/s and 3.0E-8 kg/s are chosen to represent the cases where the ventricular surface has some degrees of permeability.

**Fig. 7:**
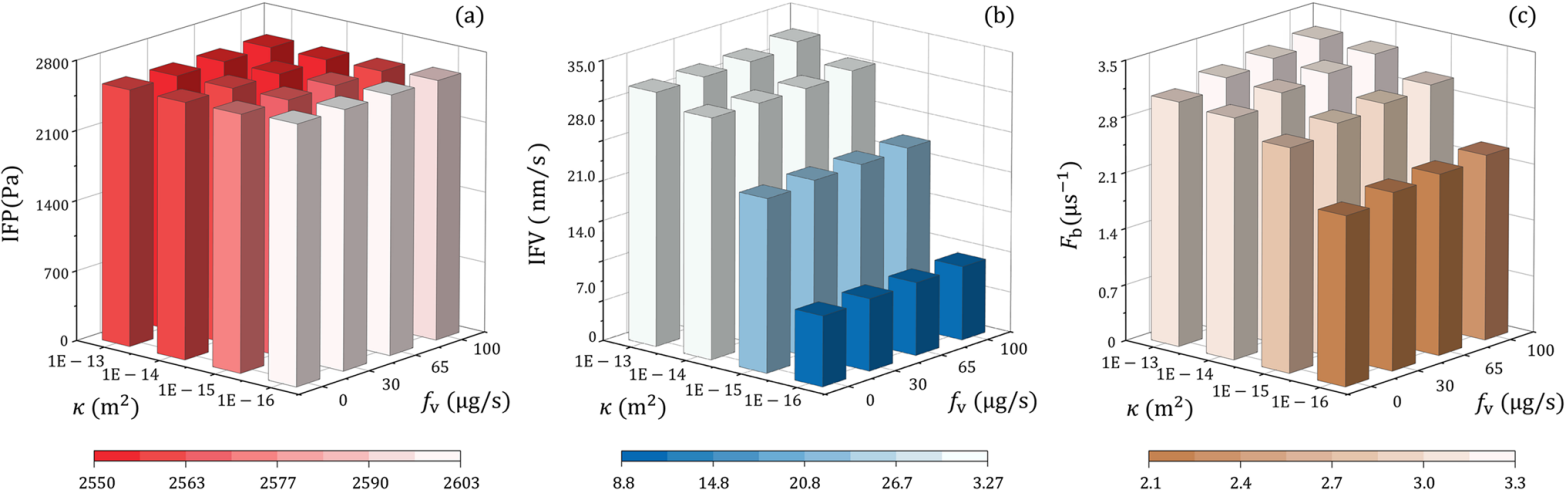
Effect of tissue permeability on the interstitial fluid flow in brain tumours with different permeable degrees of the ventricle wall. (**a**) Interstitial fluid pressure, (**b**) interstitial fluid velocity, and (**c**) fluid gain from blood.

The ISF flow in the brain tumours with different tissue hydraulic permeability and CSF flow from the ventricle are shown in Fig. 7. The similar IFP in all the tumours indicates that the impact of these two influencing factors is limited. Similarly, the responses of IFV and ${F}_{\mathrm{b}}$ to the trans-ventricle CSF flow is less significant as compared to tissue hydraulic permeability. Quantitative analyses demonstrate a negative relationship between IFP and the CSF flow from the ventricle. However, this CSF flow is positively related to IFV and ${F}_{\mathrm{b}}$, respectively.

**Fig. 8:**
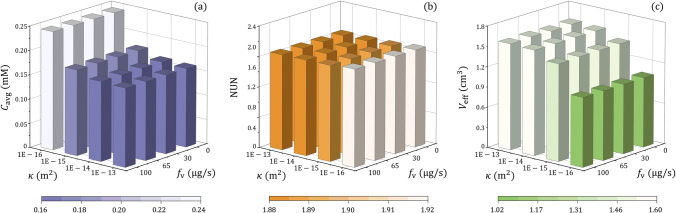
Effect of tissue permeability on the delivery outcomes in brain tumours with different permeable degrees of the ventricle wall. (**a**) Spatial-averaged concentration, (**b**) non-uniformity, and (**c**) effective distribution volume.

Figure 8 summarises the delivery outcomes in response to the changes in tissue hydraulic permeability and CSF flow from the ventricle. It is found that this CSF flow has less significant effects on the drug delivery outcomes when compared to tissue hydraulic permeability. Quantitative analyses show that the CSF from the ventricle makes distribution slightly uniform, which has the potential to expand the effective distribution volume for killing tumour cells.

### Cross-Influence with Drug Type

A variety of anticancer drugs are now used clinically to treat brain tumours, including temozolomide (TMZ), paclitaxel (PTX), and carmustine (BCNU). In addition, doxorubicin (DOX) also has the ability to kill brain tumour cells (87). Therefore, these four drugs are selected to examine the effect of tumour tissue permeability on the performance of CED.

**Fig. 9:**
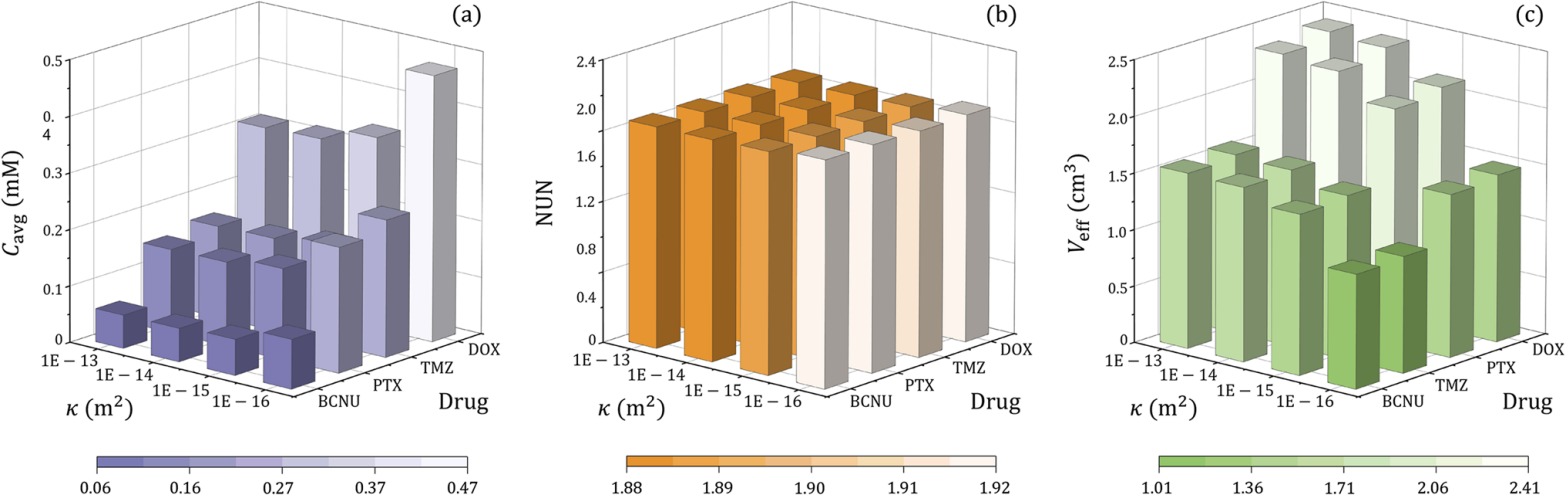
Effect of tissue permeability on the delivery outcomes of different drugs. (**a**) Spatial-averaged concentration, (**b**) non-uniformity, and (**c**) effective distribution volume.

The responses of different chemotherapy drugs to hydraulic permeability of tumour tissue are compared in Fig. 9. The higher concentration is obtained in the less permeable tumours, regardless of drug type. However, the distribution of all four drugs would be slightly heterogeneous in the tumour with lower tissue hydraulic permeability. This implies that the drugs would concentrate in limited regions, resulting in inadequate drugs to effectively kill cells in most tumour tissues. The effective distribution volume decreases with the reduction of tissue hydraulic permeability of all four drugs.

## DISCUSSION

The hydraulic permeability of tumour tissue is determined by the tissue microstructure and compositions, particularly the fibre network and polysaccharides in the extracellular matrix. Standing for the tissue resistance to the fluid flow, the low tissue hydraulic permeability leads to high intratumoural pressure and slow interstitial fluid flow upon CED infusion. Such a hydraulic environment can consequently inhibit the fluid gain from the blood and generate a more heterogeneous drug distribution. Although this distribution pattern allows achieving more localised drug delivery, the drug penetration into deep tumour tissue is simultaneously reduced. This would further lead to inadequate drug concentration in the tumour region, which is distant from the infusion site, reducing the treatment effectiveness.

CED improves drug delivery outcomes by enhancing the interstitial fluid flow in the tumour ECS. As the sources of ISF in the brain, the fluid gain from the blood and CSF flow from the ventricle contribute to a favourable hydraulic environment that improves the delivery outcomes in the tumour. Notably, the understanding of some underlying mechanisms involved in the complex brain ISF flow remains controversial. For instance, water was reported to be able to cross the BBB through different means including diffusion and vesicular transport (7, 88). This was based on the findings that the water transporting pores including Aquaporin 4 (AQP4) are located in the endfeet of astrocytes (89, 90), whereas the endothelium carries no AQP4 transporters (91). Moreover, this transvascular fluid transport is expected to be more significant in brain tumours as over 100 nm pores were found on the tumour microvasculature wall (92). On the contrary, the BBB is also reported as an impermeable barrier to fluid flux (93). Therefore, further efforts are needed to fill the knowledge gaps.

CSF heavily participates in the intracerebral ISF flow. Entering the brain parenchyma from the brain surface next to the subarachnoid space, CSF travels in the perivascular spaces alongside the arteries and eventually leaves the brain tissues through perivenous spaces, perineural spaces and lymphatic vessels in the meninges (7, 93). This physiological process is simulated in this study by applying the subarachnoid pressure to the brain surface (85). Consequently, this CSF flow is driven by the pressure difference between the brain surface and tissue IFP. It is important to point out that due to the large difference in size, microvasculature (in μm) is considered to be distributed in the brain (in cm). Therefore, the vessel geometry is not represented explicitly in this macroscale transport-based model, where the entire brain is accommodated. For in-depth analysis, a microscale model will need to be developed with the geometry of each anatomical structure represented explicitly. Specifically, the model should include the perivascular space, astrocyte endfeet, endothelial cell layer and smooth muscle cell layer. Moreover, AQP4 is a key protein involved in water transport in the brain. As its function is based on the interactions between molecules and atoms, computational models such as molecular dynamics can be applied to explore the mechanisms. Given these are out of the scope of the current study, such simulations are not included.

The results from this study show that the four chemotherapy drugs present a similar trend when responding to the changes in hydraulic permeability of tumour tissue. On the contrary, the delivery outcomes vary considerably between the drugs, depending on the properties of the drugs. One of the essential factors is drug elimination due to blood drainage. TMZ and BCNU have been applied in the clinical treatments of brain cancer because of their ability to cross the BBB upon routine intravenous administration. However, as drugs are directly infused into the tumour ECS upon CED, this transvascular transport would enhance the drug loss by blood drainage, thereby reducing delivery outcomes. In contrast, plain DOX is rarely used in routine chemotherapy to treat brain tumours, as it is usually believed unable to penetrate BBB (61). Whereas, this nature in turn lowers the drug loss to the blood in the CED treatment, enabling more DOX to be retained in the tumour. These findings suggest the important role of drug properties in determining the effectiveness of CED treatment, highlighting the demand for selecting the appropriate drugs for CED and developing the corresponding administration protocols.

**Fig. 10:**
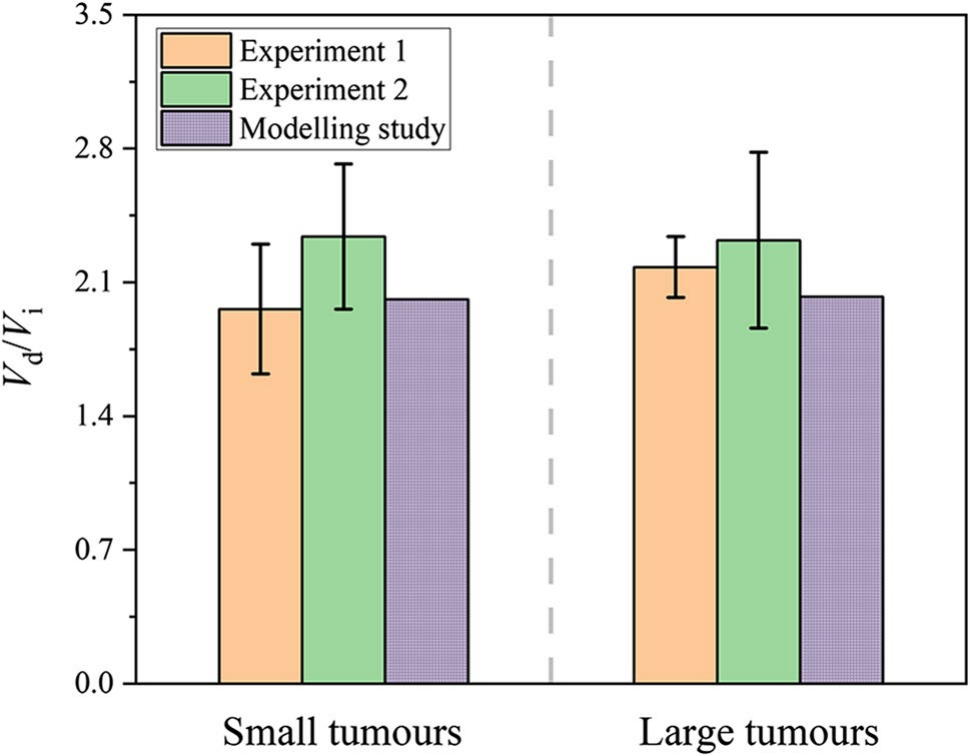
Comparison of modellingpredictions with experimental data. In the experiments, 20 μL of ‘brain penetrating’ nanoparticles were infused into the small brain tumours (~ 5 mm^3^) and large brain tumours (80 ~ 100 mm^3^) in 30 min. The nanoparticles were ~ 74 nm in diameter and stable (94). ${V}_{\mathrm{i}}$ is the total infusion volume and ${V}_{\mathrm{d}}$ is nanoparticle distribution volume.

Figure 10 compares the modelling predicted delivery outcomes with the experimental measurements under the same infusion settings (94). The *in vivo* experiments demonstrated that the tumour size has a limited impact on the distribution volume of nanoparticles, which is further confirmed by the modelling study. The predictive power of the transport-based model for predicting drug delivery to solid tumours has been validated in several reported studies using *in vivo* experimental data. The IFV in the tumour was predicted as 0.17 μm/s (22) that was well located in the experimental range from 0.13 to 0.2 μm/s (95). The predicted IFP was 1500 Pa in a realistic tumour model reconstructed from MR images (28); This pressure was in the range of 587 to 4200 Pa measured in the experiments (96). The modelling predictions on the time course of nanoparticle distribution upon CED quantitatively agree with the data from animal experiments (40), while the spatial distribution of CED-infused small molecules obtained by modelling can match the measurement using MR imaging in a qualitative manner (97). The lack of accurate models of complex delivery processes and the absence of model parameters for tissue and drug properties are two major barriers to drug delivery modelling. These limitations will be even more pronounced for those drugs that undergo complex bioreactions *in vivo*. Therefore, mathematical modelling can only provide qualitative analysis. For improvement, specific models can be developed to describe a certain drug delivery process based on the findings from biochemical studies. Microscale research (98) and the application of advanced medical imaging techniques (99) can support the determination of drug and tissue properties.

In this study, the volume fraction of intracellular space is differentiated between the cells in normal brain tissue in terms of the cell types. The calculations in Table III are based on the current reported population ratio of different types of cells. It is important to point out that these ratios could vary depending on location. This is mainly because current experiments are less able to count the number of each type of cell in the entire brain. The reported data was mainly based on small tissue specimens that were collected from certain locations in a brain, limiting the representativeness of the measurements. Cell distribution is another factor. For instance, the cell bodies of neurons mainly concentrate in the grey matter, while axons are in the white matter. However, the influences of these factors on the modelling predictions in this study are expected to be less obvious. This is because the delivery outcomes of CED are highly localised. The drug concentration in normal brain tissue, therefore, is several orders lower than in the brain tumour where the infusion catheter is located, as shown in Table V. The cell type and cell distribution could play a more significant role when drugs are delivered to the brain parenchyma, such as in the treatment of Alzheimer's disease and Parkinson’s disease.

There are several key assumptions and limitations in this study. (I) The microvasculature distribution can be highly heterogeneous, depending on the tumour type, location and stage. Since there is a lack of *in vivo* data available, blood vessels are assumed to be homogeneously distributed (36, 38). This assumption can be relaxed by using dynamic contrast-enhanced MR images (25). (II) A general model is applied to describe the nanoparticle cell uptake. It is critical to note that this cell uptake process is highly nanoparticle-specific. For instance, liposomes are able to be absorbed. However, several polymer nanoparticles enter the cell interior through the connection of different types of ligands to the receptors on the cell membrane. So that, the number of ligands on the nanoparticle surface and the non-/occupied receptors on the cell membrane will play an important role. This process is also determined by the nanoparticle dimension. For instance, ligands such as transferrin can be attached to the nanoparticle surface to enhance this process (100). However, lipid nanoparticles of 120 nm have been found unable to be endocytosed (101). Moreover, the nanoparticle cell uptake may require energy (102). Given its complexity, a specific model needs to be developed when a particular type of nanoparticle is studied. (III) Specific values of flow flux are imposed on the brain ventricle surface to represent different permeable degrees of this surface to the CSF flow. This is a simplified model as the permeability of the ventricle surface is not available. Supported by further experimental measurements of this tissue property, a porous media model can be developed for the ventricle surface to predict this CSF flow.

## CONCLUSIONS

The effects of hydraulic permeability of tumour tissue on convection-enhanced delivery of nanoparticle-encapsulated drugs to brain tumours have been examined using mathematical modelling. Results show that drugs can transport deep into a permeable tumour with a relatively uniform distribution. This is beneficial for achieving more effective cell killing in a larger tumour region, although the spatial-averaged concentration would be slightly low. The delivery outcomes of CED are more sensitive to the changes in tissue hydraulic permeability and blood pressure as compared to the cerebrospinal fluid flow from the ventricle. High blood pressure and permeable ventricle surface allow more fluid to transport from the blood and ventricle to the brain, respectively. These fluid gains can further enhance the intracerebral ISF flow, and thereby improve the delivery outcomes. Moreover, different drugs present similar responses to the changes in hydraulic permeability of tumour tissue. Results obtained from this study can deepen the understanding of the transport mechanisms involved in drug delivery to brain tumours upon CED.
